# News and Notes

**Published:** 1902-03

**Authors:** 


### *NEWS AND NOTES.*

(We are indebted to the *Medical Age* for many of these notes.)

---

THE *Atlanta Medical and Surgical Journal* and *Southern Medical Record* having been consolidated into the ATLANTA JOURNAL-RECORD OF MEDICINE, exchanges will please be sent only to the latter. We are getting many duplicates.

---

THE general health in Havana is at present considered excellent.

---

BUBONIC plague is said to be epidemic among the natives of the Fiji Islands.

---

AT the present time there are about 130,000 physicians in the United States.

---

CONSUL LANE, at Smyrna, has cabled the State Department that the bubonic plague has broken out in Asia Minor.

---

DR. WALTER B. EMERY has been elected secretary of the Atlanta Society of Medicine, to succeed Dr. Claude A. Smith, resigned.

---

THE Halcyon and the Atlanta Retreat have combined to form the Halcyon-Retreat and now occupy the Halcyon Sanatorium building.

---

PROFESSOR VIRCHOW, the eminent pathologist, who was injured while alighting from a street-car some time ago, is growing worse.

CANCER causes nearly five thousand deaths annually in London, according to the report of the medical officer of health for that city.

---

ON January 23 last the new lying-in hospital, which J. Pierpont Morgan has built on Second Avenue in New York City, was opened.

---

THE census statistics of the United States show that the proportion of deaths to population has decreased ten per cent. in the past decade.

---

THE milk commissioner of New York has ordered that hereafter smooth-faced men only will be employed for milking cows and delivering milk.

---

IT is claimed that hydrophobia does not exist in Great Britain, owing to the stringent rules of the Board of Agriculture ordering the muzzling of dogs.

---

A BILL has been introduced into the State Council of St. Louis making obligatory the reporting to the Health Board all cases of pulmonary tuberculosis.

---

DR. H. P. GAYLORD, director of the New York State Cancer Laboratory of Buffalo, has been elected a foreign member of the German Cancer Investigation Committee.

---

SENATOR PLATT has introduced a bill into Congress for the establishment of a home for lepers in the United States. A location will be selected in a warm, dry climate.

---

DR. HUNTER P. COOPER has been appointed Surgeon of the Nashville, Chattanooga and St. Louis Railroad, for the Western and Atlantic Division, between Atlanta and Chattanooga.

DR. ANNIE L. SAWYER, pleasantly remembered as the head nurse of the St. Joseph's Infirmary, has graduated in medicine in Philadelphia, and entered upon the practice of medicine here.

---

IN 1888 the Mexican National Board of Health introduced the Pasteur treatment of hydrophobia into Mexico; since that time 4,000 persons have been treated, with a total mortality of three per thousand.

---

A COMMITTEE of Russian medical men is said to have been organized to test the conclusions of Professor Polotebnoff, who denies that leprosy is contagious after a prolonged special research on the subject.

---

THE annex to the St. Joseph Infirmary is nearing completion. It will contain one of the best equipped operating-rooms in the South and will add greatly to the present facilities for the accommodation of patients.

---

THE Paris Academy of Medicine has awarded to Dr. Melanie Lipinski, of Warsaw, the Hugo prize of $200 for the best work on the history of medicine appearing in the French language during the past five years.

---

DR. T. EDWARD HAYES, surgeon-major of the Siamese navy for the past thirteen years, and formerly of Baltimore, has had the Royal Order of the White Elephant bestowed upon him by the King of Siam in person.

---

MEDICAL INSPECTOR P. M. RIXEY, physician to the late President and Mrs. McKinley, has been appointed surgeon-general of the navy with the rank of rear admiral. He is a Virginian, and a graduate of the University of Virginia.

THE *Medical Record* is authority for the following: "A woman sixty-five years old gave birth last week, in a New Jersey town, to a healthy girl. The woman's husband is seventy years old, though both are said to be young-looking for their years, and the couple had passed forty years in a childless wedlock."

---

SURRA, the disease caused by the parasite trypanosoma evansi, which affects mules, horses and camels, is reported, says an exchange, as having invaded the Philippines and making great havoc among the horses and mules. In Manila alone the quartermaster's department has lost three hundred horses within four months. The disease was at first diagnosed as glanders.

---

ONE of the most recent discoveries of Professor Hensen, the German State marine biologist, is of bacteria which keep the sea fresh by attacking the surplus organic matter in it. Other researches in Plankton show that in some places the sea is a mass of liquid food, which fish and birds inhale, as it were. Even around the arctic and antarctic poles this minute life exists in such a quantity as to permeate and color the sea.—*The Nineteenth Century and After.*

---

THE President of the Republic of Nicaragua, as a recognition of the services rendered by the American minister, William Lawrence Merry, in the interests of the construction of the Isthmian canal through Nicaraguan territory, has ceded, says *American Medicine*, to the United States land on the Island of Ometepe equal in area to one hundred city lots. This land will be known as Mount Merry, and will be devoted exclusively to hospital purpose. The gift becomes effective so soon as work is started upon the canal.

---

ACCORDING to an exchange the study of Prussian vital statistics recently published seems to show a relation of some sort between divorce, insanity and suicide. Out of a million persons, 348 divorced women committed suicide as compared with 61 married women. Three hundred and eighty-six married men committed suicide as compared with 2,834 divorced ones. In the insane asylum in Wurttemburg a similar study shows 3,024 persons who have been divorced as compared with 283 married persons, 460 bachelors and maids, and 672 widows and widowers.

---

AS a further means of avoiding septic poisoning during operations, a special mask is now being used, says an exchange, by a number of surgeons throughout Europe. The invention is the outgrowth of the fact that a great obstacle to thorough antisepsis is occasioned by the danger arising from the breath of the surgeon and his assistants. The mask is of fine gauze and tightly covers the mouth and nose, but does not interfere with the sight, hearing, or breathing. If the protection is as great as is claimed, this mask will soon become an important antiseptic precaution in all operations.

---

AN autopsy made at the Johns Hopkins Hospital recently upon the body of a negro who died after a stroke of paralysis showed, according to the *Philadelphia Medical Journal*, the transposition of the man's internal organs. The heart was entirely on the right side of the median line; the liver was mostly on the left; the spleen was transposed to a spot several inches from its normal position; while the kidneys had changed places. The stomach was far out of its natural position. This transposition of viscera was probably congenital, and may indirectly have caused death by superinducing hemiplegia.

---

DR. J. B. MATTISON, of Brooklyn, has offered a prize of $400 for the best paper on the subject, "Does the Habitual Subdermic

Use of Morphine Cause Organic Disease? If so, What?" The contest will be open for two years from December 1, 1901, to any physician, in any language. The award will be determined by a committee consisting of Dr. T. D. Crothers, Hartford, Conn., editor *Journal of Inebriety*, Chairman; Dr. J. M. Van Cott, Professor of Pathology, Long Island College Hospital, Brooklyn; and Dr. Wharton Sinkler, Neurologist to the State Asylum for the Chronic Insane, Philadelphia. All papers must be in the hands of the chairman by or before December 1, 1903. They will become the property of the American Association for the Study and Cure of Inebriety, and be published in such journals as the committee may select.

---

UNDER the name of "muscle-bed," says *American Medicine*, a device has been invented by Dr. William Anderson, of the Yale gymnasium, for testing in the horizontal human body the distribution of the blood-supply under the effect of thought and exercise, and of ascertaining the center of gravity. This apparatus rests on very accurately made knife edges, and is sensitive to the slightest pressure; it is furnished with levels, graduated scales, and indicator for recording. A body perfectly balanced on the sensitive knife edges of the muscle-bed will be affected by additional weight on either side of the point of equilibrium, causing the head to settle if the flow of blood is in that direction, or the feet to lower if the flow is toward them. In the case of a subject balanced on the muscle-bed who was told to answer some question requiring thought, although not a muscle was moved the rush of blood to the head caused by the mental effort created a change of the center of gravity.

---

A MISSION which deals with over 2,000 cases every year, has a finely equipped hospital steamer, and has built two hospitals on the Labrador shore, is erecting, according to an Eastern contemporary, a third hospital in northern Newfoundland. All the medical attention received by the people along these coasts is given by the physicians belonging to the mission. There are so few physicians, however, in comparison to the vast territory to be covered, that many requiring it do not receive sufficient medical attention. Among the natives sanitation is totally disregarded, and isolation for contagious diseases is practically unknown. In making a tour of the coast the physicians found cases of poisoned wounds, eye diseases, dangerous digestive troubles, scurvy, cancer, and tuberculosis that had never known medical treatment. Besides the medical work the mission has been instrumental in establishing coöperative stores, providing work, finding homes for orphans, and relieving many cases of destitution.

---

GREAT dissatisfaction, says *American Medicine,* is expressed over the meager pay of hospital stewards of the army. At present they receive but $30 a month; a full steward receives $45. The duties of the stewards and acting stewards are to look after and distribute hospital stores and supplies; to care for hospital property; to compound and administer medicines; to supervise the preparation and serving of food; to maintain discipline in the hospital and watch over its general police; to prepare the hospital reports and returns; to supervise the duties of the hospital corps in hospital and in the field. The steward must be an efficient disciplinarian, expert clerk, accurate arithmetician, and a trustworthy pharmacist, with as much knowledge of materia medica, therapeutics, and minor surgery as will enable him to give sound advice and suitable treatment in the minor ailments and accidents; in addition, he must have that higher knowledge for use in the wards which enables the experienced nurse to appreciate the condition of those who are seriously ill. The pay is considered entirely inadequate to the services required.

DR. J. D. COAKLEY, of Chicago, is now engaged in establishing an exclusive association to be formed only of those physicians and surgeons who have made original and extensive investigations which have added much to scientific knowledge. While essentially American, membership will be drawn from all parts of the world. Dr. Coakley, who has just returned from Europe, has given the names of a number of foreign medical men interested in the association. Among these are Dr. Renvers of Berlin, Dr. Hallion of Paris, and Drs. Starling and Foster of London. All applicants for membership must be at least twenty-five years old, graduates of a school of medicine and surgery recognized by the International Medical Association, and affidavits must be presented to show the originality of work and the practical and satisfactory results of experiments. Members less than forty years old will be required to perform satisfactory experiments on living animals on such lines as will be in harmony with their particular line of work. This must be continued each year for ten years, at the end of which time he may become an honorary member of the congress. Less work will be required of those more than forty years old. The different divisions of the congress will meet every year for the next five years with their respective national associations, and every three years with the International Medical Association, after which time the meeting place will be selected by a majority vote. The last clause of the by-laws states the object of the congress to be "the furthering of scientific research, honest work, and honest reports, all of which, it is to be hoped, will result in the further alleviation of suffering and the prolongation of life." Before any line of work will be accepted as complete, the investigator will be compelled to demonstrate his results before a committee, to be selected by the president of the congress.—*Philadelphia Medical Journal.*

